# Lymph Node Metastasis in Head and Neck Squamous Cell Carcinoma: Evolving Prognostic Markers, Molecular Insights, and Implications for Precision Staging †

**DOI:** 10.3390/diagnostics16060855

**Published:** 2026-03-13

**Authors:** Andrés Coca-Pelaz, Ehab Y. Hanna, Orlando Guntinas-Lichius, Luiz P. Kowalski, Juan Pablo Rodrigo, Robert P. Takes, Marc Hamoir, Remco de Bree, Francisco J. Civantos, K. Thomas Robbins, Carlos Suárez, M. P. Sreeram, Karthik Rao, Alfio Ferlito

**Affiliations:** 1 Department of Otolaryngology, Hospital Universitario Central de Asturias, University of Oviedo, ISPA, IUOPA, CIBERONC, 33011 Oviedo, Spain; acocapelaz@yahoo.es (A.C.-P.); jprodrigo@uniovi.es (J.P.R.); 2 Department of Head & Neck Surgery, The University of Texas MD Anderson Cancer Center, Houston, TX 77030, USA; eyhanna@mdanderson.org; 3 Department of Otorhinolaryngology, Jena University Hospital, 07747 Jena, Germany; orlando.guntinas@med.uni-jena.de; 4 Head and Neck Surgery and Otorhinolaryngology Department, A C Camargo Cancer Center, Sao Paulo 01509-010, Brazil; lp_kowalski@uol.com.br; 5 Head and Neck Surgery, Faculty of Medicine, University of Sao Paulo, Sao Paulo 01246-903, Brazil; 6 Department of Otolaryngology, Head and Neck Surgery, Radboud University Medical Center, 6525 GA Nijmegen, The Netherlands; r.takes@kno.umcn.nl; 7 Department of Otolaryngology, Head & Neck Surgery, St Luc University Hospital, King Albert II Cancer Institute, 1200 Brussels, Belgium; marc.hamoir@saintluc.uclouvain.be; 8 Institut de Recherche Expérimentale et Clinique (IREC), UCLouvain, 1200 Brussels, Belgium; 9 Department of Head and Neck Surgical Oncology, University Medical Center Utrecht, 3584 CX Utrecht, The Netherlands; r.debree@umcutrecht.nl; 10 Department of Otolaryngology-Head and Neck Surgery, Sylvester Comprehensive Cancer Center, University of Miami, Miami, FL 33136, USA; fcivanto@med.miami.edu; 11 Department of Otolaryngology Head Neck Surgery, SIU School of Medicine, Southern Illinois University, Springfield, IL 62702, USA; kthomasrobbins@gmail.com; 12 Faculty of Medicine, University of Oviedo, 33003 Oviedo, Spain; csuareznieto@gmail.com; 13 Department of Head and Neck Oncology, Sri Shankara Cancer Foundation, Bangalore 560004, India; drsreeram111@gmail.com (M.P.S.); karthik.nag.rao@gmail.com (K.R.); 14 International Head and Neck Scientific Group, 35100 Padua, Italy

**Keywords:** head and neck cancer, squamous cell carcinoma, lymph node metastasis, extranodal extension, prognosis, survival

## Abstract

Lymph node metastasis (LNM) is one of the most powerful prognostic determinants in head and neck squamous cell carcinoma (HNSCC). The extent and pattern of nodal involvement critically influence staging accuracy, therapeutic decision-making, and patient outcomes. However, the biological and clinical implications of nodal disease remain complex and continue to evolve. We aim to synthesize current clinical and translational evidence regarding the prognostic and therapeutic impact of LNM in HNSCC and to highlight emerging trends relevant to precision staging. A narrative review was conducted through a structured literature search in PubMed and Scopus (2008–2025), with emphasis on studies published in the last five years. Meta-analyses, large cohort studies, and evidence-based guidelines addressing prognostic factors, biological mechanisms, and management strategies were critically appraised. LNM is consistently associated with reduced overall and disease-free survival across major head and neck subsites. Key independent prognostic variables include the number of metastatic nodes, extranodal extension, and involved cervical levels. Recent advances, such as refinements in the AJCC 8th edition, sentinel lymph node mapping, high-resolution imaging, and molecular profiling, have improved early detection and refined risk stratification. LNM remains central to prognostic evaluation and treatment selection in HNSCC. Integrating biological insights with molecular diagnostics and advanced imaging will be essential to achieving precision staging and individualized therapeutic strategies.

## 1. Introduction

Head and neck squamous cell carcinoma (HNSCC) remains a substantial global health burden, with more than 900,000 new cases and nearly 470,000 deaths annually, according to recent GLOBOCAN estimates [1]. Despite improvements in multimodal therapy, long-term survival gains have been modest, largely due to regional recurrence and distant metastasis [2].

Among prognostic factors, lymph node metastasis (LNM) represents one of the most decisive determinants of outcome in HNSCC. Several studies indicate that the presence of regional nodal spread can reduce 5-year survival rates by approximately 50% compared with patients without nodal disease [3]. However, this prognostic significance differs notably according to the tumor’s molecular profile, with a more limited impact in HPV-positive cases [4]. The anatomical complexity and rich lymphatic drainage of the head and neck region predispose tumors to early dissemination via lymphatics [5,6,7,8].

However, nodal disease is highly heterogeneous in its clinical implications. Beyond mere presence, the extent, number, size, location (lower neck lymph nodes), and pathological features of nodal metastasis influence prognosis and therapeutic decision-making. For instance, extranodal extension (ENE), defined as the extension of tumor cells beyond the lymph node capsule into perinodal soft tissues, has emerged as a potent adverse prognostic marker and is now incorporated into recent staging refinements [9,10,11]. In fact, the 8th edition of the American Joint Committee on Cancer (AJCC)/the Union for International Cancer Control (UICC) TNM staging system gives specific emphasis to ENE in nodal classification, with distinct criteria applied to HPV-positive and HPV-negative disease [9,11,12]. These refinements underscore the increasing importance of incorporating biological features into staging beyond purely anatomical parameters.

Meta-analyses and large cohort studies confirm that ENE positivity is independently associated with worse locoregional control, higher distant metastasis rates, and reduced overall survival (OS), particularly in HPV-negative HNSCC [9,10,13]. Up to now, ENE can only be definitively diagnosed by surgery, and imaging has been found to be notoriously inaccurate at predicting ENE [14,15]. Given its prognostic significance, radiological prediction of ENE (via advanced imaging or AI) is an area of active investigation [9,16,17,18,19,20,21].

In surgically treated patients, another layer of prognostic nuance arises from lymph node yield (LNY), defined as the number of lymph nodes removed, and the lymph node ratio (LNR), representing the ratio of positive lymph nodes to total dissected nodes. Higher nodal yield may improve staging accuracy and capture occult disease, while LNR offers a continuous metric of disease burden [22].

Advances in imaging, sentinel lymph node biopsy (SLNB), artificial intelligence, and radiomics have further refined nodal assessment, enabling improved detection and characterization of nodal disease. Emerging radiomic approaches may contribute to improved preoperative prediction of extranodal extension, representing a potential bridge between imaging biomarkers and biologically informed staging [16,23,24].

Despite these advances, key controversies and knowledge gaps endure. The optimal management of clinically negative necks (N0) in early-stage disease remains debated, particularly with respect to elective neck dissection (END) versus observation, as well as the diagnostic role of SLNB for accurate staging. The role of de-escalation protocols in HPV-positive oropharyngeal cancer, especially in relation to ENE risk, remains under active investigation. Moreover, the integration of biomarkers (molecular, genomic, and microenvironmental) with imaging and clinical risk stratification is nascent but promising [25,26,27]. It is also essential to differentiate HPV-positive and HPV-negative disease, as their lymphatic dissemination patterns, biological aggressiveness, and prognostic significance differ markedly [28].

Emerging systemic therapies, including immunotherapy, further highlight the biological complexity of nodal disease and the importance of understanding the tumor–immune microenvironment [29,30,31,32].

Together, these developments highlight a progressive transition from purely anatomical staging toward multidimensional, biologically informed models of nodal assessment. Given the central importance of nodal metastasis in prognosis and treatment planning, this narrative review aims to present an up-to-date, integrative synthesis of current evidence on the biological mechanisms, prognostic implications, and clinical management of LNM in HNSCC. Through a critical lens on recent advances, we seek to identify unresolved issues and propose directions for future research. Beyond summarizing existing evidence, this review aims to conceptualize lymph node metastasis within an integrated precision-staging framework, linking tumor biology, nodal microenvironment, advanced imaging, quantitative nodal metrics, and clinical decision-making.

## 2. Methodological Approach

This article was conceived as a narrative review, designed to integrate and critically discuss the current evidence regarding the prognostic and clinical significance of LNM in HNSCC. A structured literature search was performed in PubMed and Scopus (January 2008–March 2025) using combined MeSH terms and free-text keywords: “head and neck squamous cell carcinoma”, “lymph node metastasis”, “extranodal extension”, “nodal yield”, “lymph node ratio”, and “prognosis”. Additional keywords included “artificial intelligence,” “radiomics,” and “deep learning”. Additional relevant studies were identified through manual citation tracking from retrieved articles and recent meta-analyses. The search strategy combined MeSH terms and free-text keywords using Boolean operators (e.g., “head and neck squamous cell carcinoma” AND “lymph node metastasis” OR “extranodal extension” OR “lymph node ratio” OR “sentinel lymph node biopsy”). The initial search yielded several thousand records. Titles and abstracts were screened to identify clinically relevant studies, prioritizing recent meta-analyses, large cohort studies, and high-quality translational research.

We included peer-reviewed original studies, clinical trials, large retrospective cohorts, systematic reviews, and evidence-based guidelines relevant to the scope of this review. Articles were selected based on clinical relevance, methodological quality, and contribution to understanding the biological mechanisms, prognostic value, or therapeutic implications of nodal disease. Non-English publications, case reports, and commentaries without primary data were excluded.

The collected evidence was analyzed to identify major domains of relevance, including biological pathways of lymphatic spread, prognostic impact of nodal characteristics (number, size, and ENE), and evolving clinical management strategies. Particular emphasis was placed on studies published in the last five years, reflecting rapid advances in imaging, molecular pathology, and risk stratification. To ensure methodological rigor and transparency, the review followed the SANRA (Scale for the Assessment of Narrative Review Articles) criteria as a quality framework [33]. The application of SANRA criteria aimed to enhance structure, transparency, and critical appraisal, while preserving the integrative and hypothesis-generating nature of a narrative review. SANRA is a validated tool designed to enhance the methodological rigor of narrative reviews through six domains: justification of the article’s importance, statement of concrete aims, adequacy of literature search, appropriate referencing, scientific reasoning, and presentation quality. Each domain was considered during article selection, synthesis, and manuscript drafting to ensure transparency and reproducibility. This review aims to provide a comprehensive, balanced, and up-to-date synthesis of the literature most relevant to the clinical and prognostic dimensions of LNM in HNSCC. No ethical approval was required, as only previously published data were analyzed.

## 3. Epidemiology and Biological Basis of Nodal Metastasis

The epidemiology and biological basis of LNM in HNSCC are critical determinants of disease progression and patient prognosis [34]. LNM is a highly significant negative prognostic factor, with regional involvement reducing OS by 30–50% [35].

### 3.1. Clinicopathological Risk Factors

Perineural Invasion (PnI) and lymphatic/vascular invasion (LI/VI) are important pathological features. PnI is consistently identified as a major adverse prognostic factor in HNSCC. A meta-analysis of over 101 studies, including 26,062 oral squamous cell carcinoma (OSCC) patients, demonstrated that PnI is associated with significantly worse OS (HR = 1.45, 95% CI 1.32–1.58), DSS (HR = 1.87, 95% CI 1.65–2.12), and DFS (HR = 1.82, 95% CI 1.69–1.96) compared with PnI-negative patients [36,37]. Therefore, PNI is a marker of biologically aggressive disease, and its presence is independently associated with higher rates of nodal involvement, ENE, and adverse survival outcomes [38,39,40]. In a population-based cohort of 745 OSCC patients, LI was present in approximately 15.4%; VI in approximately 3.1%. LI was strongly correlated with higher grade, nodal involvement, advanced stage, and resulted in significantly lower 5-year OS (41.1% vs. 66.8%) and recurrence free-survival (RFS) (38.3% vs. 59.7%) compared with LI-negative cases [41]. Similarly, in 127 patients with tongue SCC, PnI and LI/VI combined were independent predictors of worse OS [42].

Also, PnI/LI/VI double positivity in 98 patients T3-4 OSCC was associated with elevated rates of nodal metastasis, distant metastasis (rate, 33.3%), and notably reduced 5-year DSS (27.7%) compared with single or double negative cases [43].

The degree of tumor differentiation is a risk factor for LNM. Poor differentiation has been associated with an increased risk of LNM in laryngeal carcinoma [44]. In OSCC, poor differentiation (G3) was associated with reduced RFS (HR: 1.75) [45]. In OPSCC, lower pathological differentiation significantly increased the likelihood of cervical LNM [6]. Poorly differentiated SCC of the oropharynx has a risk of cervical LNM 11 times that of well-differentiated SCC (OR = 10.929, 95% CI 1.546–77.264) [46]. In OSCC, higher tumor grading increased the risk of occurrence of occult LNM 2.7-fold (OR = 2.68) [47].

### 3.2. Tumor-Related Factors

Several intrinsic characteristics of the primary tumor, distinct from clinicopathological risk factors, influence the likelihood and extent of nodal metastasis and subsequent patient prognosis. Among these, depth of invasion (DOI) has emerged as one of the most robust predictors of lymph node metastasis.

Depth of Invasion (DOI) is recognized as a primary risk factor for LNM in laryngeal carcinoma [35] and is an independent risk factor for occult LNM in early OSCC [48]. Depth of invasion (DOI) and tumor thickness represent distinct parameters; DOI is defined relative to the reconstructed mucosal baseline and incorporated into AJCC 8th edition staging, whereas tumor thickness reflects absolute vertical measurement. DOI is closely related to local recurrence and metastasis in OSCC and is an important indicator for predicting LNM [49,50]. The risk of occult LNM guides the selection of neck management in early OSCC. Based on a decision analyses in 1994, END is typically advised when the estimated risk exceeds 20% [50]. However, with improved staging, more selective neck dissections (less morbidity), other quality of life estimates (by patients and not surgeons), improved follow-up and salvage treatments, the outcome of an updated decision analysis would likely differ [51]. For OSCC, a tumor depth of 5 mm is often suggested as a threshold for occult LNM, indicating the need for END [52,53,54,55]. The risk of occult LNM may be higher in T3 and T4 OSCC tumors due partly to the greater DOI [52]. The risk of occult LNM can be reduced to below 5% when the neck is staged using sentinel lymph node biopsy (SLNB) [23], which allows for a more accurate detection of micrometastases and obviates the need for elective neck dissection in selected early-stage OSCC cases in which the neck does not need to be opened for resection or reconstruction [56]. A more detailed discussion of micrometastasis and isolated tumor cells is provided in Section 7.2. The sensitivity for detecting occult lymph node metastasis appears comparable between SLNB and END [57]. Moreover, randomized clinical trials and a network meta-analysis have shown that a cN0 management strategy based on SLNB had similar survival outcomes as an END strategy in early-stage OSCC patients [58,59,60].

Micrometastases and ITCs have also been reported in early-stage, clinically node-negative OCSCC. Their prognostic relevance remains uncertain, as most available evidence comes from small retrospective series with heterogeneous methods of detection. While some authors suggest that micrometastatic disease may represent an intermediate biological state between true nodal negativity and overt metastasis, current data do not support altering management solely on the basis of MM or ITCs [61]. Previous commentaries have emphasized the need for caution, noting that neither MM nor ITCs have demonstrated consistent impact on survival or local–regional control to date [56,62]. A recent expert review highlighted the emerging relevance of micrometastasis and ITCs in refining nodal staging in OCSCC, emphasizing advances in detection techniques (including step-serial sectioning, immunohistochemistry, and molecular profiling), while noting that their clinical impact remains uncertain and requires prospective validation [63].

### 3.3. Mechanisms of Lymphatic Dissemination in HNSCC

HNSCC commonly disseminates through lymphatic drainage pathways [64]. Lymph nodes serve a paradoxical role: they are pivotal sites for initiating specific immunity (crucial for maintaining antitumor immune response) yet also serve as primary conduits for tumor metastasis [22,65].

Key mechanisms and processes involved in lymphatic dissemination and LNM include:

- Specific lymphatic mechanisms: Metastasis primarily occurs through the invasion or co-option of lymphatic vessels, with LNM being a crucial indicator of disease progression [22,34].
- Tumor microenvironment and molecular factors: LNM involves complex processes such as the formation of pre-metastatic niches, lymphangiogenesis, and epithelial–mesenchymal transition (EMT) [5,66,67,68]. EMT facilitates detachment and migration through loss of epithelial adhesion and acquisition of mesenchymal traits. Formation of premetastatic niches involves tumor-derived exosomes and stromal remodeling that prime lymphatic microenvironments for colonization. Immune dysfunction within draining lymph nodes, including T-cell exhaustion and altered antigen presentation, further contributes to metastatic progression [69,70,71].
- Immune compromise: Lymph node colonization can potentially induce systemic immune dysfunction, thereby promoting tumor progression and distant metastasis [65,72].

Recent evidence links nodal immune exhaustion and PD-L1 expression within metastatic lymph nodes to poorer outcomes and potential responsiveness to immunotherapy, suggesting that nodal immune profiling may guide future therapeutic decisions [73].

### 3.4. Regional Lymphatic Anatomy and Preferred Metastatic Pathways

Patterns of LNM in HNSCC are site-specific and generally follow predictable lymphatic drainage routes (Table 1). Metastasis to lymph nodes is often characterized by primary, secondary, or tertiary levels of involvement, based on frequency. Levels II and III are considered primary drainage levels for all major subsites discussed (oral cavity, oropharynx, larynx, hypopharynx) [74].

Skip metastases, defined as LNM occurring in downstream neck levels without involvement of the expected first-echelon nodes, are relatively uncommon in laryngeal SCC compared with certain oral and oropharyngeal subsites. Large pattern-mapping and cohort studies indicate that laryngeal tumors, particularly glottic and transglottic primaries, typically follow more predictable drainage pathways affecting levels II–IV, while true skip metastases are reported only infrequently (usually low single-digit percentages) [77,78]. Supraglottic lesions show higher overall rates of nodal involvement than glottic tumors, but even in supraglottic series, the incidence of isolated skip metastasis remains modest and is often linked to advanced T-stage or atypical lymphatic anatomy [79,80]. By contrast, oral tongue and floor-of-mouth primaries have denser, multidirectional lymphatic networks and a higher propensity for unpredictable or “skip” patterns of spread, as shown in meta-analyses and series of OSCC [81,82]. Understanding these subsite-specific differences is essential for tailoring END and radiation target volumes.

### 3.5. Incidence of LNM by Subsite and Stage

Across HNSCC, occult LNM occurs in approximately 20–30% of clinically node-negative (cN0) cases [47,83], reaching up to 40–50% in OSCC. In cohorts undergoing END for cN0 OSCC, the LNM rate was 31.3% in one study [84]. The risk of occult LNM is often categorized by T-classification: T1 and T2 tumors have a rate of occult LNM of about 20–30% [85]. T1N0 patients have a pooled sentinel node positivity (a proxy for occult LNM) of 26%. T2N0 patients have a pooled sentinel node positivity of 35% [86]. In the cN0 OSCC cohort of Rose et al. [86], LNM rates by T-stage were: 22% (T1), 25% (T2), 38% (T3), and 67% (T4).

In patients with OPSCC, a retrospective analysis from a regional cohort in Shanxi, China, reported a cervical LNM rate of 89.2% [6]. However, such elevated incidence may reflect specific population or referral characteristics, as large registry-based analyses (e.g., SEER and NCDB datasets) generally report lower overall rates. HPV-positive OPSCC is nonetheless characterized by a high propensity for early lymphatic dissemination. In tonsillar SCC, the overall prevalence of occult contralateral LNM was approximately 10%, with rates increasing from 8% in cT1–T2 tumors to 19% in cT3–T4 tumors. Stratification by ipsilateral nodal status shows contralateral occult LNM rates of about 1% for ipsilateral N0 disease and 12% for ipsilateral N+ disease.

In the larynx, malignant tumors of supraglottic origin show a greater tendency for locoregional involvement compared with glottic or subglottic carcinomas [35]. Early-stage glottic lesions (T1) rarely metastasize to regional lymph nodes. For cN0 supraglottic SCC, the pooled risk of occult isolated contralateral/bilateral LNM was 7.49% [87]. Risk of occult contralateral/bilateral metastasis in cN0 supraglottic SCC was stratified by stage/status, for T1-T2 patients, the risk was 5.18% (95% CI: 2–64%; I 2 = 81%), and 8.73% (95% CI: 5.92–12.69%, I 2 = 0%) for T3–T4 patients. In patients with confirmed ipsilateral pN+ status, the risk increased to 21%.

The heterogeneity of reported occult metastasis rates across subsites and T-categories reflects both biological variability and methodological differences in staging techniques. Such variability complicates universal thresholds for elective neck treatment and highlights the need for risk-adapted diagnostic strategies.

## 4. Prognostic Impact of Lymph Node Metastasis

LNM constitutes the most critical prognostic factor in HNSCC, signifying disease progression and an inherently poor prognosis [88]. The presence of regional LNM is widely associated with a substantial reduction in OS, estimated at up to 50% [89], and correlates directly with lower DFS and a need for more aggressive treatment modalities [90].

### 4.1. Relationship Between Nodal Status and Survival Metrics

The prognostic stratification capabilities of lymph node status extend beyond simple presence or absence (N+ vs. N0) to incorporate quantitative metrics that reflect the true burden of metastatic disease [91]. Studies focused on laryngeal SCC demonstrate a clear inverse relationship between increasing pathological N (pN) stage and long-term survival outcomes [89]. For instance, 5-year DSS and DFS rates decrease progressively across pathological N stages (e.g., pN0: DSS 81%, DFS 72%; pN1: DSS 70%, DFS 62.5%; pN3b: DSS 34%, DFS 30%) [35].

### 4.2. Influence of the Number of Affected Lymph Nodes

The absolute number of positive lymph nodes (NPLN) has emerged as a particularly robust prognostic factor, independently associated with mortality and recurrence risk [91]. In HNSCC, mortality risk continuously escalates with an increasing NPLN count. A large-scale analysis using the Surveillance, Epidemiology, and End Results (SEER) database indicated that the NPLN might be superior to both the LNR and the conventional AJCC N staging system for stratifying prognosis in surgically treated HNSCC patients [92]. This emphasis on nodal count is supported by quantitative studies showing that mortality risk increases sharply with each additional metastatic node, particularly beyond four [91]. Similarly, in laryngeal SCC, survival decreases sharply based on NPLN, with a dramatic drop observed for counts of 6–10 nodes (DSS 55%, DFS 35%) and those exceeding 20 nodes (DSS 16%, DFS 2%) [35]. Furthermore, analyzing data from three prospective randomized controlled trials (NRG/RTOG 9501, NRG/RTOG 0234, and EORTC 22931) demonstrated significant differences in OS and DFS when HNSCC outcomes were stratified based on a change point of ≤5 versus >5 positive lymph nodes [93]. Besides number, the location of LNM also has prognostic value: low jugular lymph node metastasis is associated with a higher risk of distant metastasis and thus survival [94].

### 4.3. Role of LNR and ENE

The LNR, calculated as the ratio of positive lymph nodes to the total number of excised lymph nodes, is also recognized as an important prognosticator [89]. LNR is often considered a reliable metric because it simultaneously accounts for both the disease burden and the quality of the neck dissection performed [95,96]. A meta-analysis confirmed that higher LNR values confer a worse prognosis in HNSCC, demonstrating increased hazard ratios for OS (HR 1.96), DFS (HR 2.43), and DSS (HR 2.07) [22]. In OSCC, LNR has been identified as a robust and consistent prognostic factor, outperforming the absolute number of metastatic nodes in some studies, particularly showing that an LNR >3.4% is significantly associated with increased recurrence and reduced OS [84,97,98,99,100,101]. Specifically, for advanced laryngeal and hypopharyngeal malignancies, an LNR of >0.05 acts as an independent adverse prognostic factor for both recurrence-free interval (RFI) and OS [89]. Given the heterogeneity of reported LNR cut-off values across anatomical subsites, a comparative summary of the most commonly proposed thresholds is provided in Table 2. This comparative overview highlights the substantial heterogeneity of proposed LNR thresholds across subsites and underscores the need for standardized definitions in future staging models.

Importantly, the wide variability in reported LNR cut-off values likely reflects both biological heterogeneity across subsites and methodological differences in surgical extent, pathological processing, and statistical modeling. In oral cavity SCC, relatively low thresholds (0.03–0.07) appear sufficient to stratify risk, whereas in hypopharyngeal and laryngeal cancers, higher cut-offs are often required to discriminate prognosis. This suggests that LNR does not represent a universally transferable parameter but rather a subsite-dependent metric that must be interpreted within specific anatomical and biological contexts [22]. For advanced laryngeal SCC, the Log Odds of Positive Lymph Nodes (LODDS), defined as the logarithmic ratio between the number of positive and negative lymph nodes, has also been proposed as a potentially superior prognosticator compared with LNR and NPLN for DFS and OS [102]. LODDS provides mathematical advantages by incorporating both positive and negative lymph node counts in a logarithmic scale, allowing improved risk stratification, particularly among patients with low or absent nodal positivity (pN0), where traditional ratios may lack discriminatory power. Moreover, recent population-based and multicenter analyses suggest that LODDS may outperform traditional nodal metrics across multiple head and neck subsites, demonstrating consistent prognostic value for OS even beyond laryngeal cancer [106,107,108].

ENE represents a critical and dominant prognostic factor in HNSCC [109,110]. ENE is indicative of aggressive tumor biology and is strongly correlated with increased risk of locoregional recurrence, distant metastasis, and decreased survival [111]. ENE is such a strong predictor that its presence typically mandates escalation of adjuvant treatment (i.e., the addition of chemotherapy to radiotherapy) to improve survival metrics [35,112].

The extent of ENE also holds quantifiable prognostic value. Some series differentiate between microscopic ENE (≤2 mm) and macroscopic ENE (>2 mm), the latter being associated with worse outcomes and guiding the need for adjuvant chemoradiotherapy [111]. Comparative analysis in OSCC and OPSCC highlighted the prognostic dominance of ENE: 5-year survival rates drastically differed between non-metastasized tumors (N0, 67%), intranodal disease (N+R−, 59%), and ENE (N+R+, 31%) [113]. Furthermore, ENE has been confirmed as an independent predictor of worse RFI (HR 3.53) and OS (HR 2.44) in advanced laryngeal and hypopharyngeal carcinoma [89]. These findings collectively establish ENE as a pivotal variable for both prognostication and adjuvant treatment decision-making. A comparative overview of the major nodal prognostic markers, their biological basis, and clinical implications is summarized in Table 3, which synthesizes the biological basis, prognostic impact, and clinical implications of the principal nodal markers currently proposed for precision nodal staging. Important controversies persist, particularly regarding the prognostic weight of ENE in HPV-positive disease and the clinical relevance of traditional cutoff thresholds [111]. Although ENE consistently confers adverse prognosis in HPV-negative HNSCC, its relative impact appears attenuated in HPV-positive OPSCC, where overall survival remains comparatively favorable despite nodal burden. This biological divergence underscores that identical anatomical nodal features may carry distinct prognostic implications depending on viral status, supporting the need for biologically contextualized staging rather than uniform anatomical classification [19,78]. However, in a multicenter cohort study, it was found that in patients with OSCC, adjuvant chemotherapy is beneficial in patients with major ENE (>2 mm), but may not be beneficial in patients with minor ENE (<2 mm) [114].

### 4.4. Comparison Between TNM Classifications (7th vs. 8th Edition)

The AJCC staging system provides the standard framework for HNSCC [122], but the 8th edition introduced significant revisions to enhance prognostic accuracy [123].

The most critical modification in the 8th edition was the incorporation of ENE into the N category for most HNSCC subsites (excluding HPV-related oropharyngeal cancer) [3]. Clinically, the presence of ENE results in a classification of cN3b. Pathologically, ENE also shifts classifications, notably elevating some smaller metastatic nodes into higher N categories (e.g., pN2a includes a single ipsilateral lymph node ≤3 cm with ENE+) [124].

Despite these adjustments, studies suggest that the traditional size, number, and laterality criteria used in the 7th edition and the inherited framework of the 8th edition often fail to adequately stratify patients [92]. Survival curves for groups defined by AJCC N classification (N1, N2, N3) frequently demonstrate overlap in OS for oral cavity, oropharynx and hypopharynx primary sites [74]. For instance, a multicenter study focusing on laryngeal cancer found no statistically significant separation in survival outcomes between pN1 and pN2 disease in the 8th edition, suggesting that quantitative metrics like LNR and NPLN were superior for patient stratification [89]. Furthermore, research indicated that the level of LNM (e.g., primary, secondary, tertiary drainage groups) provides prognostic information independent of the traditional AJCC N classification for OSCC, laryngeal SCC, and OPSCC, suggesting that nodal level can refine existing staging tools [74].

**Figure 1:**
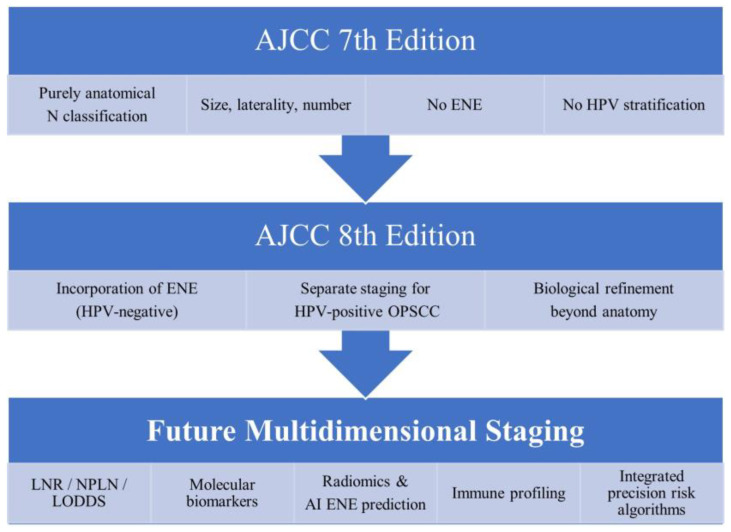
Evolution of Nodal Staging in HNSCC: From Anatomical Classification to Precision Nodal Staging.

The 8th edition notably introduced a separate staging system for HPV-positive (p16-positive) OPSCC, recognizing its distinct biological behavior and favorable prognosis [6]. For these tumors, the metastatic lymph node status appears to have a less severe prognostic impact compared with HPV-negative HNSCC. However, the 9th edition of the UICC (2025) is planning to extend the incorporation of ENE to HPV-positive OPSCC as well [111]. While the anticipated refinements in the UICC 9th edition aim to enhance biological accuracy, their implementation may pose challenges in non-academic or resource-limited settings, where access to advanced molecular diagnostics, quantitative nodal metrics, or AI-supported tools may be restricted. Ensuring feasibility and reproducibility across diverse clinical environments will therefore be essential before widespread adoption. The conceptual evolution of nodal staging is illustrated in Figure 1, highlighting the transition from purely anatomical classification toward multidimensional precision nodal staging models integrating biological and quantitative parameters.

Historically, lymph node size served as a primary criterion for defining nodal stage [22]. However, recent evidence suggests that the prognostic utility of size is often eclipsed by the quantitative burden of metastatic nodes [91]. Multivariable models analyzing OSCC, which adjust for the number of metastatic nodes, found that LNM size was no longer significantly associated with mortality. In diagnostic imaging, size remains a marker, though frequently non-specific [64]. Current diagnostic guidelines suggest a minimal axial diameter of 10 mm for magnetic resonance imaging (MRI) and 12 mm for computed tomography (CT) as criteria indicative of metastasis [125].

Despite consistent evidence supporting the prognostic relevance of ENE, nodal burden, and LNR, effect sizes vary substantially across subsites and HPV status. For example, the relative hazard associated with ENE appears attenuated in HPV-positive OPSCC compared with HPV-negative disease, suggesting that biological context modifies the impact of anatomical nodal parameters. These discrepancies underscore the limitation of uniform staging approaches across heterogeneous tumor entities. Collectively, these findings suggest that current TNM staging, while clinically pragmatic, incompletely captures the multidimensional biological heterogeneity underlying nodal metastasis. The persistence of survival overlap across N categories indicates that anatomical descriptors alone are insufficient to fully explain prognostic variability [92]. Importantly, integration of quantitative and molecular nodal parameters into future TNM editions would require consensus-driven harmonization of definitions, thresholds, and measurement methodologies. Incorporation into formal staging systems is unlikely to occur without robust evidence demonstrating incremental prognostic value beyond existing N categories. Therefore, precision nodal staging should initially function as a complementary risk stratification layer rather than a replacement for TNM classification [124].

## 5. Clinical and Therapeutic Implications

### 5.1. Surgical Management: Selective, Radical, and Extended Cervical Dissection

Surgical intervention, primarily neck dissection (ND), serves both diagnostic and therapeutic roles. The type of ND performed depends primarily on the tumor site and nodal status, including selective neck dissection (SND), modified radical neck dissection (MRND), and radical neck dissection (RND) [84,115,126].

For clinically node-negative (cN0) disease, the decision for END is crucial; it is often recommended when the risk of occult LNM exceeds 20% [84]. In OSCC patients with cN0 neck, the initial surgical approach typically involves SND addressing levels I-III, sometimes including Level IV [51]. In early-stage OSCC patients, SLNB is a valid alternative to SND [26,58,59,60].

In terms of surgical quality and prognosis, the LNY is often cited, with benchmarks frequently set at ≥18 nodes [116,117,118,119]. However, the prognostic value of a rigid LNY threshold (e.g., 18 nodes) is challenged by recent studies in OSCC, some of which found no significant survival benefit for patients with ≥18 nodes retrieved [84]. These discrepancies likely reflect variation in surgical technique, pathological processing, and institutional practice patterns rather than purely biological differences. Consequently, LNY may function more reliably as a quality-of-care surrogate marker than as an intrinsic biological prognostic variable [116]. For definitive guidance, the minimum optimal number of lymph nodes is recommended to be >10 for SND and >14 for MRND in OSCC, according to the National Cancer Grid Head & Neck Cancer Management Guidelines (2019) [127] and supported by prior surgical trials [95]. The absence of universal consensus regarding optimal lymph node yield thresholds further underscores the need for standardized pathological and surgical reporting.

For laryngeal and hypopharyngeal carcinomas, SND of levels II–IV is the standard approach in cN0 patients. In the presence of nodal metastasis (cN+), MRND is reserved for cases with extensive or bulky nodal disease, fixed nodes, or suspected ENE. Importantly, even in many cN1 and selected cN2 patients, SND remains appropriate and oncologically safe, provided adequate nodal clearance is achieved [89,126]. Furthermore, for tumors with high potential for contralateral spread, particularly midline or near-midline tonsillar SCC, base of tongue tumors, and selected supraglottic primaries, contralateral neck management should be considered, rather than assumed routinely. The decision is primarily guided by tumor laterality, depth of medial extension, and T-stage [87,128]. For cT3/T4 tonsillar SCC, the prevalence of occult contralateral LNM reaches 19% [128]. In cN0 supraglottic SCC, contralateral neck dissection is recommended for tumors with Type C lymphatic drainage patterns (i.e., predominant bilateral lymphatic outflow), as these carry a higher likelihood of occult contralateral metastasis, or when ipsilateral nodal metastasis is confirmed pathologically [87]. Standardizing surgical reporting and nodal yield metrics may facilitate inter-institutional comparison and quality assessment.

### 5.2. Radiotherapy and Chemoradiotherapy: Adjuvant Indications Based on Nodal Involvement

The identification of adverse pathological factors (APFs) following surgery dictates the need for adjuvant therapy, aimed at reducing the risk of locoregional recurrence [109,110]. Adjuvant concurrent chemoradiotherapy (CTRT) is widely accepted as the standard of care for two major indications: the presence of positive surgical margins and the presence of ENE [129]. ENE is recognized as a strong and highly significant prognostic factor, making adjuvant treatment with CTRT essential to improve DSS and DFS. The use of adjuvant CTRT has been shown to significantly improve DSS and DFS outcomes compared with surgery alone in retrospective analyses, particularly when balancing the initial worse disease stage of patients requiring multimodality treatment [17,129,130]. A common regimen for adjuvant concurrent chemotherapy includes Cisplatin 100 mg/m^2^ once every three weeks (optimal option) or Cisplatin 30–40 mg/m^2^ weekly [127,131]. A recent randomized clinical trial showed that CRTCT with weekly cisplatin is noninferior to 3-weekly cisplatin for patients with postoperative high-risk locally advanced HNSCC, but probably has less toxicity [131].

Beyond these critical factors, the overall nodal burden influences adjuvant planning:

• Node positivity (pN+): Adjuvant radiotherapy is recommended in cases with multiple positive lymph nodes, ENE, or additional high-risk features. In contrast, for isolated pN1 disease without ENE, PnI, or LI/VI, the indication for postoperative RT remains controversial and may be individualized based on surgical completeness and multidisciplinary assessment [35].
• Multiple positive nodes: The number of involved lymph nodes is an established prognostic factor, with survival progressively decreasing as nodal burden increases. However, the indication for adjuvant CTRT is not determined by nodal count alone. Pooled analyses of the landmark EORTC 22931 and RTOG 9501 randomized trials demonstrated that the survival benefit of postoperative CTRT is confined to patients with ENE and/or positive surgical margins, rather than those with multiple positive nodes in isolation [132,133]. Therefore, in the absence of ENE or involved margins, adjuvant radiotherapy alone may be appropriate even when several lymph nodes are involved, with treatment individualization guided by overall nodal burden and patient-specific risk stratification [91].
• Prognostic metrics: The quantitative metrics of NPLN and LNR show prognostic value independent of traditional pN staging and should be incorporated into the decision paradigm for adjuvant treatments in laryngeal SCC. Higher LNR values are associated with poorer prognosis, justifying more aggressive regional and systemic adjuvant approaches [22,93,134,135].

High nodal burden (i.e., multiple metastatic lymph nodes) remains a strong prognostic indicator of recurrence and decreased survival. However, escalation to adjuvant CTRT is primarily justified when high nodal burden coexists with ENE or positive surgical margins, rather than nodal count alone. Future prospective, ideally randomized studies are needed to determine whether elevated nodal burden or specific lymph node ratio thresholds should independently inform postoperative treatment intensity. A risk-adapted treatment selection model integrating quantitative and biological nodal parameters may further refine postoperative decision-making. For example, patients with low NPLN (≤2), low LNR (<0.05), and absence of ENE may be considered for de-intensified adjuvant radiotherapy protocols, whereas patients with high LNR (>0.15), major ENE, and unfavorable immune signatures may benefit from intensified systemic therapy or enrollment in clinical trials exploring novel combinations. Such stratification would move beyond binary ENE-based escalation toward multidimensional risk modeling [28,132,133].

### 5.3. Sentinel Lymph Node Biopsy: Emerging Role in Early Stages

SLNB represents a valuable, minimally invasive approach to managing the cN0 neck in early-stage OSCC (cT1-2N0), balancing the risk of occult LNM against the morbidity of END [48]. SLNB positivity is considered a strong predictor of (additional) occult LNM, alongside DOI [136,137,138]. The pooled positivity rate for SLNB in early OSCC (T1N0 and T2N0) is approximately 26.7% [86]. When successfully performed, SLNB accurately stages the regional lymph nodes for T1–T2 OSCC. However, the accuracy and widespread applicability of SLNB remain reliant on specialized technical expertise and experience [127].

An emerging application for SLNB involves assessing recurrence risk in salvage settings, specifically for second primary or recurrent OSCC, even in patients who have previously undergone radiation or ND. In these complex cases, lymphatic mapping has demonstrated a high negative predictive value (NPV) of approximately 97%, suggesting its utility in guiding salvage management [139]. Another advantage of staging the neck using SLNB is the detection of micrometastases and ITCs, easily missed during routine histopathological examination of the neck dissection specimen [63].

### 5.4. De-Escalation Strategies Based on Nodal Burden

The LNR, the NPLN, and the LODDS can help identify patients with low-risk nodal involvement who may be candidates for de-escalation of adjuvant therapy [35]:

• pN1 disease: In OSCC, a single positive lymph node in the absence of ENE, LI/VI, PnI, or close/positive margins carries a more favorable prognosis than multiple-node involvement. Accordingly, the benefit of routine postoperative radiotherapy in patients with isolated pN1 disease remains debated, and several guidelines allow for selective omission of adjuvant RT in this scenario, provided that an adequate neck dissection has been performed and no other adverse pathological features are present. Conversely, when additional high-risk features are identified, adjuvant radiotherapy (and CTRT in the presence of ENE or involved margins) remains indicated [140,141,142].
• OPSCC (HPV-related): Due to generally favorable outcomes, the observation that survival may not significantly change until a higher number of positive nodes (up to 5 in one analysis) is reached suggests potential candidates for de-intensification protocols currently under investigation [92]. Furthermore, in patients with pN2a, HPV-negative HNSCC with pathologic ENE, recent evidence indicates that the addition of chemotherapy to adjuvant radiotherapy may not significantly improve overall survival [143]. Given that these findings diverge from current clinical guideline recommendations, further prospective studies are warranted to validate the omission of chemotherapy in this subset of patients. Nevertheless, the apparent tolerance of higher nodal burden in HPV-positive disease should be interpreted cautiously, as emerging evidence suggests that certain high-risk features—such as major ENE or very high nodal counts—may partially mitigate the favorable biology associated with HPV-mediated tumors. This highlights the complexity of balancing de-escalation strategies against residual biological risk [17,21]

### 5.5. Impact on Follow-Up and Surveillance Protocols

Follow-up and surveillance protocols must be tailored based on disease risk and nodal burden to detect recurrence early [27].

#### 5.5.1. Risk-Stratified Follow-Up

The presence of occult LNM in OSCC patients is associated with significantly worse progression-free survival compared with nodal disease detected preoperatively [47]. This elevated risk suggests that patients with occult LNM may require a more aggressive and stringent follow-up protocol [144,145]. However, progression-free survival and overall survival for patients with occult metastases may not differ significantly from those with nodal disease detected preoperatively, provided that surgical management is optimal and adjuvant therapy is appropriately applied [146]. Using strict ultrasound-guided fine needle aspiration citology surveillance by experienced radiologists, survival is not negatively influenced in early-stage OSCC patients. Using a ‘wait and scan’ follow-up strategy instead of elective neck treatment, unnecessary neck dissection and its accompanying morbidity can be avoided in the vast majority patients. However, for the small proportion of patients with delayed metastases, more extensive treatment with adjuvant radiotherapy will be needed [147]. The overall goal of surveillance is risk assessment, leading to individualized strategies [35]. Nomograms, which are personalized predictive tools incorporating multiple factors (e.g., TNM, age, and smoking status), are increasingly used to achieve higher accuracy in prognosis and thus guide tailored surveillance intensity [105,148,149,150,151,152,153]. Future surveillance paradigms may incorporate integrated imaging–molecular monitoring strategies. For instance, in patients with high-risk nodal features (e.g., major ENE or elevated LNR), periodic ctDNA assessment combined with AI-assisted radiologic interpretation could allow earlier detection of minimal residual disease. Conversely, in biologically low-risk nodal profiles, reduced imaging frequency may be justified, minimizing cost and radiation exposure without compromising oncologic safety. Such adaptive surveillance models would represent a practical extension of precision nodal staging into longitudinal care [1,12,27].

#### 5.5.2. Surveillance Modalities

Standard post-treatment surveillance relies on routine clinical examination supplemented by advanced imaging techniques [127]:

• High-risk imaging: For patients at high risk for distant metastasis (e.g., N3 nodes, multiple bilateral nodes, lower cervical nodes, T4b primary, or advanced hypopharyngeal cancer), PET-CT or contrast-enhanced CT (CECT) of the thorax is generally recommended [154,155,156]. For T3 and T4 nasopharyngeal tumors, PET-CT/MRI should optimally be performed annually for 5 years [76].
• Imaging interpretation: Radiologic follow-up uses structured reporting systems (like NIRADS) to categorize the suspicion of recurrence. A high suspicion (Category 3), indicated by a new or enlarging discrete nodule/mass or a morphologically abnormal lymph node, necessitates a biopsy. Low suspicion (Category 2) warrants short-interval follow-up (e.g., 3 months) or repeat PET imaging [157,158].

## 6. Limitations

This review is narrative in nature and may therefore be subject to selection and publication bias. Although we applied transparent and systematic search criteria, the lack of quantitative synthesis limits the ability to establish causal inferences. Nevertheless, this approach enabled a comprehensive integration of recent translational and clinical evidence relevant to LNM in HNSCC.

## 7. Toward Precision Nodal Staging: Integrated Future Directions

The rapid advances in oncology necessitate the continuous refinement of prognostic tools and diagnostic modalities to improve risk stratification and guide treatment decisions in HNSCC [22,124].

### 7.1. Integrated Biological and Diagnostic Framework

The limitations of the current AJCC N staging system are evident, particularly the overlap in survival outcomes among subgroups like pN1 and pN2 disease in laryngeal SCC [22,35,104]. Future prognostication within precision nodal staging relies on incorporating quantitative measures of tumor burden.

The LNR and the NPLN are superior prognostic markers to traditional pN staging in HNSCC, correlating well with patient OS and DFS. High NPLN counts (over five) are linked to increased mortality [35,84,92]. Emerging metrics like the LODDS may offer even greater predictive capability for DFS and OS in advanced laryngeal SCC compared with LNB and LNR [102].

ENE is a crucial, negative prognostic factor already included in the AJCC 8th edition nodal classification for most HNSCC sites [89,159]. Current evidence indicates that the presence of ENE is also associated with significantly decreased OS, DSS, and DFS compared with ENE-negative cases, even in the context of HPV-mediated disease, suggesting that this parameter must be incorporated in the N staging also in HPV-positive disease [17,18,19,20,21].

The evaluation of lymph nodes in HNSCC is shifting from an anatomic to a molecularly driven paradigm. Traditional staging, based on size and number of involved nodes, inadequately reflects the biological diversity that determines metastatic behavior and treatment response. Recent transcriptomic studies have shown that LNMs harbor distinct molecular subtypes such as immune, invasive, and proliferative, each associated with different prognoses and patterns of failure [160]. These signatures, absent in the corresponding primary tumors, suggest that nodal profiling may provide superior prognostic information and guide tailored therapeutic strategies [161].

Integrative proteogenomic and epigenetic studies have further refined this understanding [162]. Multi-omic analyses of tumors, lymph nodes, and body fluids have revealed that immune modulation, myeloid activation, and epithelial–mesenchymal transition (EMT) pathways underpin nodal dissemination. Specific genetic alterations, such as ARHGAP15 mutations and widespread DNA hypomethylation, distinguish occult metastases from truly node-negative disease [1]. These insights herald the potential for molecular risk prediction and noninvasive detection of nodal involvement offering a means to personalize elective neck treatment and reduce unnecessary morbidity.

At the same time, tumor-draining lymph nodes are emerging as critical sites of immune priming [163]. Their indiscriminate removal or irradiation may compromise systemic antitumor immunity and limit responses to immunotherapy. The future of lymph node evaluation thus lies in coupling molecular diagnostics with immune profiling, preserving immunologically active nodes while targeting those biologically primed for metastasis. In this molecular era, lymph node assessment will evolve from anatomical mapping to biologic stratification, reshaping both prognostication and the philosophy of neck management in head and neck cancer.

**Figure 2:**
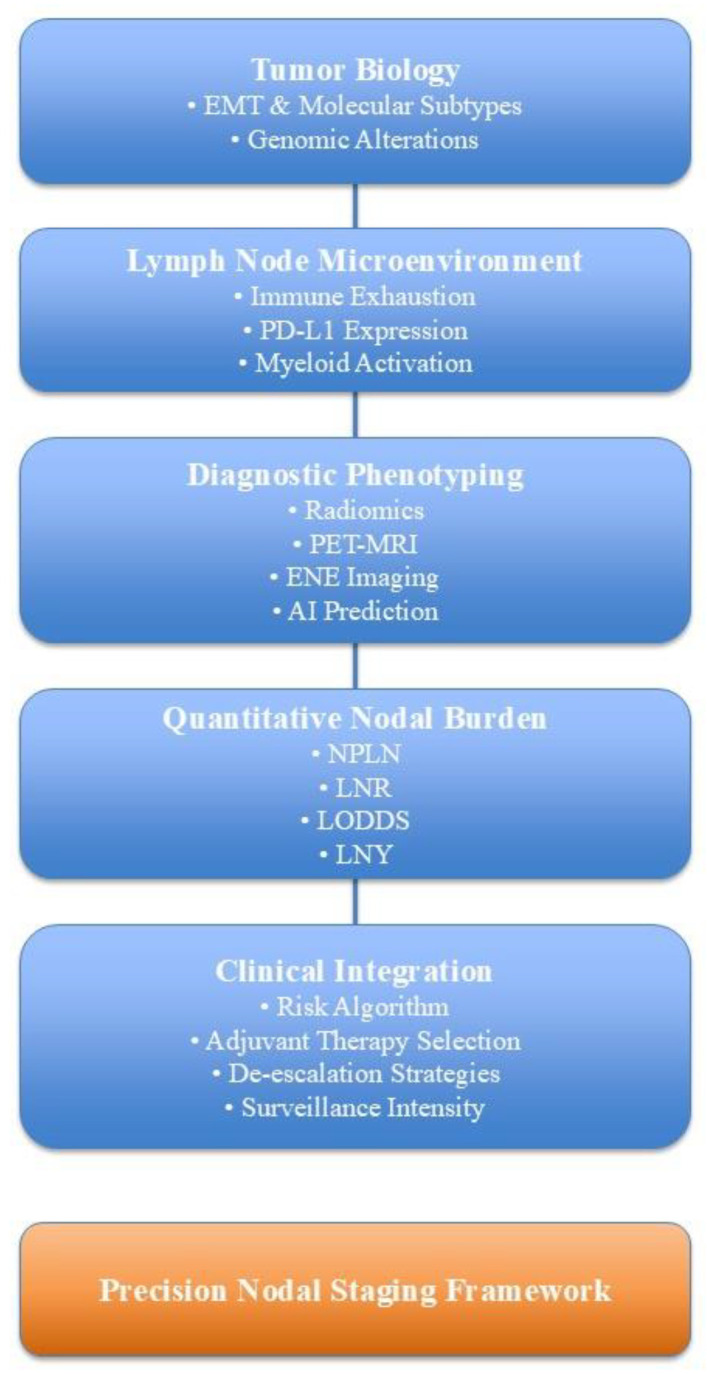
Conceptual framework for multidimensional nodal assessment in HNSCC.

Together, these parameters suggest that future nodal staging should integrate anatomical, quantitative, molecular, and immune dimensions into a multidimensional model. A conceptual integration of these interacting biological, diagnostic, and quantitative dimensions is illustrated in Figure 2, which summarizes the proposed multidimensional precision nodal staging framework. The figure illustrates the proposed transition from conventional anatomy-based nodal staging toward a multidimensional precision framework integrating tumor biology, lymph node immune microenvironment, imaging phenotypes, quantitative nodal burden metrics, and clinical decision-making algorithms.

### 7.2. Emerging Diagnostic Technologies

Advances in molecular biology aim to detect microscopic metastatic disease, such as micrometastases (0.2–2.0 mm) and ITCs (<0.2 mm), which current routine histology or imaging may miss [63,120,164,165]. Molecular techniques offer exquisite sensitivity for detecting these minute deposits [164]. Potential molecular biomarkers associated with occult LNM include gene expression profiles, circulating tumor DNA (ctDNA), miR-205, desmoglein 3 (DSG3), pan-cytokeratin (CK) AE1/AE3, HPV-16, activin-A, cyclin D1, E-cadherin, and neural progenitor lineage (NPL) [48]. Understanding the underlying mechanisms of lymphatic dissemination, such as the formation of pre-metastatic niches, lymphangiogenesis, and the role of the tumor microenvironment, remains crucial for future diagnostic and therapeutic strategies [34].

Functional and advanced imaging technologies are vital for enhancing the precision of preoperative assessments and defining the extent of nodal disease. PET/CT demonstrates superior diagnostic performance compared with conventional CT and MRI for detecting LNM in HNSCC patients [166]. Specifically, in OSCC, PET/CT exhibits the highest pooled sensitivity (0.87) and diagnostic odds ratio (DOR, 24.85), highlighting its discriminatory power [167]. By combining anatomical data with functional metabolic assessment, PET/CT enhances sensitivity and specificity, particularly useful in detecting metastatic disease that purely anatomical imaging might overlook. For patients with cN0 necks, FDG PET-CT offers high specificity and NPV for ruling out nodal involvement [83]. PET/CT is also deemed optimal for screening distant metastasis in high-risk patients (e.g., N3 nodal disease, advanced hypopharyngeal cancer) [168,169,170].

MRI plays a complementary role to CT in HNSCC staging and is particularly valuable when higher soft-tissue contrast is required, such as in evaluating perineural spread, cartilage invasion, and deep soft tissue interfaces [64]. Advanced MRI techniques, including diffusion-weighted imaging and dynamic contrast-enhanced MRI, show promise in improving the detection of occult nodal disease and refining preoperative risk assessment. Furthermore, PET/MRI combines the advantages of both modalities, offering higher diagnostic accuracy for nodal status compared to MRI alone [167,171,172]. These advanced techniques are essential for evaluating difficult features like ENE.

The integration of Artificial Intelligence (AI) represents a significant frontier in improving diagnostic precision and prognostic models for HNSCC [173]. AI has been successfully employed to classify cervical lymph nodes in HNSCC with high levels of accuracy, sometimes exceeding 90% [64,167]. Specifically, AI algorithms coupled with radiomics (the extraction of quantitative features from medical images) are promising for enhancing the accuracy of lymph node assessment, including the challenging detection of ENE [63,111]. Incorporating AI-based algorithms could also refine existing standardized reporting systems like Node-RADS [64]. Overall, integrating AI into the diagnostic workflow, risk evaluation, and prognostic assessments has the potential to enhance personalized patient care [173]. Prospective validation and integration of AI-driven models into clinical workflows will be key to ensuring their translational utility.

Nodal micrometastases and ITCs have prognostic significance in patients with HNSCC. Immunohistochemical and molecular detection methods have demonstrated that the presence of micrometastases and ITCs in lymph nodes is associated with poorer DSS and OS, as well as increased risk of locoregional recurrence, even in patients who are conventionally staged as node-negative by routine histopathology [120,121,174]. The adverse prognostic impact of micrometastases is significant and may warrant consideration of more aggressive adjuvant therapy, while isolated tumor cells appear to confer a similar risk as micrometastases in some studies [63].

However, definitions and detection methods for micrometastases (typically >0.2 mm and ≤2 mm) and isolated tumor cells (<0.2 mm) are not yet standardized, and their clinical management remains investigational [120]. Current consensus guidelines do not incorporate micrometastases or isolated tumor cells into routine staging or treatment algorithms, and further prospective studies are needed to clarify their significance and guide management.

### 7.3. Tumor–Immune Microenvironment and Therapeutic Implications

Neoadjuvant immunotherapy is increasingly being incorporated into the management of resectable HNSCC, particularly through immune checkpoint inhibition. Early phase II and III trials have demonstrated meaningful pathological tumor and nodal responses, including cases of complete sterilization of metastatic lymph nodes [175]. As a consequence, post-treatment pathological staging (ypTNM) is becoming progressively relevant, with ypN status capturing a biological continuum ranging from complete immune-mediated clearance to residual viable metastatic deposits or treatment-induced fibrosis. However, the prognostic significance of ypN in HNSCC remains insufficiently defined, and it is not yet clear how these patterns should influence indications for adjuvant therapy [176]. Future research should prioritize standardized pathological response scoring in both primary and nodal tissue, correlate ypN patterns with survival outcomes, and clarify whether ypTNM categories require adaptation in the immunotherapy era.

### 7.4. Operational Challenges and Standardization

The lack of standardized radiological reporting and pathological assessment remains a significant challenge. Standardization in pathology is essential to reliably incorporate powerful prognostic factors like LNR, NPLN, and ENE/LI/VI/PnI into clinical practice [22]. There is currently no consensus on the precise cut-off values for LNR, as evidenced by ranges reported between 0.045 and 0.20 [22,35,89,103]. Standardization must address the entire workflow, including surgical lymph node dissection technique and detailed pathological evaluation, to mitigate bias and ensure the objective calculation of metrics like LNY and LNR [22,35]. Furthermore, rigorous, consistent definitions for micrometastases and ITCs are required to allow meaningful comparison across studies. Beyond technical standardization, economic and infrastructural constraints represent significant barriers to implementation. Advanced imaging modalities (PET/MRI), radiomics pipelines, molecular profiling platforms, and circulating tumor DNA assays require substantial financial investment and specialized expertise, which may not be universally available. In low- and middle-income settings, even standardized pathological reporting of LNR or NPLN may be inconsistently implemented. Therefore, precision nodal staging models must be adaptable to variable-resource environments and allow tiered implementation strategies.

Standardization in imaging aims to improve consistency through systems like Node-RADS, which standardize the reporting of radiological findings regarding lymph node involvement using CT or MRI [177]. This approach helps address the prognostic controversy arising from heterogeneous radiological reports.

Ultimately, prospective multicenter studies are necessary to validate the predictive capacity of evolving prognostic factors such as LNR and NPLN and integrate them robustly into modernized staging systems and treatment paradigms, thus optimizing risk stratification and informing decisions about adjuvant therapies.

In addition, neoadjuvant immunotherapy further highlights the importance of preserving lymphatic immune function, as tumor-draining lymph nodes act as key sites for antitumor immune activation. Recent studies show that nodal metastases may respond differently from primary tumors to immune checkpoint inhibition, and the timing of lymphadenectomy relative to immunotherapy may influence therapeutic efficacy. As immunotherapy becomes more integrated into surgical management, treatment sequencing will require careful consideration to balance oncologic control with preservation of systemic immune competence.

Given these challenges, a structured roadmap toward clinical implementation may help translate emerging diagnostic advances into practical staging frameworks. Inter-institutional variability in surgical technique, pathological processing, radiologic interpretation, and reporting standards represents a major obstacle to comparability of nodal metrics. Without harmonized protocols, parameters such as LNR, LNY, or radiologic ENE may not be directly transferable across centers. International consensus initiatives and structured reporting templates will therefore be essential prerequisites for successful implementation of multidimensional nodal staging frameworks [11,177].

### 7.5. Roadmap Toward Clinical Implementation

The transition from conventional anatomical staging toward precision nodal staging requires progressive integration of biological, imaging, and quantitative parameters into clinical workflows. Rather than representing a single disruptive change, this evolution is likely to occur through incremental phases involving technological validation, translational integration, and eventual paradigm shifts in staging philosophy.

Near-term priorities: In the short term, several developments are poised for clinical adoption. Advances in imaging-based assessment, particularly artificial intelligence-assisted radiologic evaluation and improved prediction of extranodal extension (ENE), may enhance preoperative risk stratification. Concurrently, validation and standardization of quantitative nodal metrics, including lymph node ratio (LNR), number of positive lymph nodes (NPLN), and lymph node yield (LNY), may provide more reproducible prognostic tools that complement traditional TNM staging. Structured reporting systems and harmonized pathological definitions will be essential to facilitate widespread implementation across diverse clinical settings.

Mid-term translational integration: Over the intermediate horizon, integration of emerging molecular and immunological diagnostics may further refine nodal risk assessment. Liquid biopsy approaches, including circulating tumor DNA (ctDNA), hold promise for detecting occult nodal disease and monitoring minimal residual disease. Spatial transcriptomics and multi-omic profiling of metastatic lymph nodes may provide deeper insight into tumor–immune interactions and biological heterogeneity, enabling more precise identification of high-risk nodal phenotypes. These developments may allow stratification beyond anatomical burden toward biologically defined disease subsets. Before incorporation into routine clinical practice, emerging diagnostic tools (including AI-based imaging classifiers and molecular nodal signatures) will require prospective multicenter validation with standardized endpoints. External validation across heterogeneous populations is essential to avoid algorithmic bias and ensure reproducibility. In addition, regulatory approval pathways for AI-based clinical decision-support systems vary internationally and may impose additional requirements regarding transparency, explainability, and clinical safety. Without rigorous validation and regulatory oversight, integration into standard staging systems would remain premature [173,177].

Long-term paradigm shifts: In the longer term, nodal staging systems may evolve toward multidimensional models incorporating anatomical features, quantitative nodal burden, imaging phenotypes, molecular signatures, and immune microenvironment characteristics. Such integrated frameworks could enable adaptive treatment strategies, including personalized decisions regarding elective neck treatment, adjuvant therapy intensity, and surveillance protocols. Ultimately, the concept of precision nodal staging aims to transition from static classification toward dynamic risk modeling capable of guiding individualized management while minimizing overtreatment.

### 7.6. Emerging Therapeutic and Biological Strategies Targeting Nodal Metastasis

While advances in diagnostic stratification are important, future progress will also require strategies capable of modifying the biology of lymph node metastasis itself. Increasing understanding of the molecular and microenvironmental mechanisms governing nodal dissemination provides multiple potential therapeutic entry points [72]. Targeting lymphangiogenesis represents one promising avenue. VEGF-C/VEGFR-3 signaling plays a central role in tumor-induced lymphatic remodeling and metastatic spread. Pharmacologic inhibition of lymphangiogenic pathways may reduce nodal colonization and metastatic efficiency, although clinical validation in HNSCC remains preliminary. Equally important is modulation of the tumor-draining lymph node immune microenvironment. The nodal niche functions not merely as a passive filter but as an immunologically dynamic organ capable of either mounting anti-tumor responses or promoting immune tolerance [66,70]. Strategies aimed at reversing nodal immune suppression, including checkpoint inhibition optimization, macrophage reprogramming, and stromal targeting, may enhance regional disease control. Spatial transcriptomics and single-cell profiling have begun to reveal intranodal heterogeneity, identifying metastatic subclones and immune escape programs that may inform targeted therapeutic selection. In the future, spatially resolved molecular profiling of nodal metastases could guide individualized adjuvant strategies [162]. Liquid biopsy technologies, including circulating tumor DNA (ctDNA) and extracellular vesicle analysis, may enable earlier detection of nodal involvement or minimal residual disease, potentially refining indications for elective neck treatment and postoperative therapy [1]. Finally, preventive strategies aimed at disrupting pre-metastatic niche formation, including interference with tumor-derived exosomes, bone-marrow-derived progenitor recruitment, and stromal remodeling, represent an emerging field of translational investigation. Although still experimental, these approaches shift the focus from reactive treatment of established metastases toward proactive interception of nodal colonization. Collectively, these evolving biological strategies suggest that the future of nodal management may extend beyond improved classification toward active modulation of metastatic competence.

### 7.7. Operational Framework for Precision Nodal Staging

A practical precision staging model could integrate:

(1) Anatomical metrics (nodal size, laterality, level).
(2) Quantitative burden (NPLN, LNR, LODDS).
(3) Imaging biomarkers (radiomics-based ENE prediction, metabolic activity).
(4) Molecular signatures (immune subtype, ctDNA, EMT markers).

These components could be combined into a weighted risk algorithm designed to guide:

• Selection of elective neck dissection vs. SLNB.
• Indication for adjuvant chemoradiotherapy.
• Surveillance intensity.
• Eligibility for de-escalation protocols.

An example of a hypothetical integrated diagnostic workflow could proceed as follows: in a patient with cT2 OSCC and radiologically negative neck (cN0), advanced MRI with radiomic analysis identifies features suspicious for micro-ENE despite sub-centimeter nodes. Concurrently, elevated circulating tumor DNA (ctDNA) levels suggest occult dissemination. Although conventional TNM staging would classify this patient as low-risk, integration of imaging and molecular signals would shift management toward elective neck dissection rather than surveillance or SLNB alone. Conversely, a patient with limited nodal enlargement but low LNR, absence of ENE on imaging, and negative ctDNA might qualify for less aggressive adjuvant strategies [24].

## 8. Toward a Biologically Integrated Model of Nodal Assessment

Unlike prior reviews focusing primarily on isolated prognostic markers, this review frames lymph node metastasis within the emerging concept of precision nodal staging. We suggest that lymph node metastasis should no longer be conceptualized solely as a binary staging variable, but rather as a dynamic biological ecosystem reflecting tumor–host interaction. Integrating imaging phenotypes, quantitative nodal metrics, and molecular immune signatures may allow refinement of current TNM staging toward a multidimensional risk model.

## 9. Conclusions and Clinical Implications

LNM remains the dominant determinant of outcome in HNSCC, yet current nodal staging systems continue to rely primarily on anatomical descriptors. Accumulating evidence demonstrates that nodal burden, ENE, LNR, micrometastatic disease, molecular features, and immune contexture each provide incremental prognostic information beyond conventional N classification.

Three major conclusions emerge from this analysis. First, nodal disease should be conceptualized as a multidimensional biological process rather than a purely anatomical event. Second, quantitative nodal metrics (e.g., number of positive nodes, LNR, ENE extent) consistently refine risk stratification across subsites, although effect sizes vary according to HPV status and tumor location. Third, integration of imaging, pathological, and molecular parameters offers a realistic pathway toward precision nodal staging.

These observations generate testable hypotheses for future research:

(1) Composite nodal scores incorporating quantitative and molecular variables will outperform current TNM N categories in predicting distant metastasis and disease-specific survival;
(2) Biologically defined nodal phenotypes may identify patient subsets suitable for treatment de-escalation or intensification;
(3) Early interception of pre-metastatic niche formation could alter the natural history of regional disease.

Priority research directions include prospective multicenter validation of composite nodal models, harmonization of quantitative reporting standards, incorporation of AI-assisted imaging biomarkers, and integration of spatial and liquid biopsy-derived molecular data into clinical workflows. Ultimately, the evolution from anatomical staging toward biologically informed nodal classification is not merely a refinement of prognostic stratification, but a necessary step toward truly precision-based management of regional disease in HNSCC.

## Figures and Tables

**Table 1:** Preferred metastatic pathways according to primary site.

Primary Site	Primary/Preferred Metastatic Pathways (Ref)	Additional Notes on Spread (Ref)
**OSCC**	Levels I, II, and III [74,75]	Involvement of nodal level IV or V portends a worse prognosis than disease limited to levels I–III [75]
**OPSCC**	Levels II and III (ipsilateral internal jugular nodes), and retropharyngeal nodes [64,74]	OPSCC originating from the palatine tonsils and base of the tongue is more likely to metastasize to cervical LNs. High propensity for bilateral metastases in midline lesions, base of tongue primaries, and T4 tumors [6]
**Larynx-General**	Levels II–IV [35]	Levels I and V are affected at very low percentages, almost always with simultaneous involvement of levels II–IV [35]
**Larynx-Supraglottic**	Levels II–III and IV (frequently bilateral for median lesions and T3-T4 glottic tumors; preferentially ipsilateral for lateral lesions) [35]	The supraglottic origin has a greater tendency for locoregional involvement compared with glottic or subglottic carcinoma. Bilateral spread may occur, especially in midline or advanced lesions (T3–T4) [35].
**Larynx-Subglottic**	Level VI (pre- and paratracheal lymph nodes) [35]	Anatomical connections exist between lymphatic networks of the hemilarynx at the supraglottic and subglottic levels, providing an anatomical prerequisite for possible metastasis to the contralateral cervical lymph nodes [35]
**Larynx-Glottic**	Levels II–III and IV (ipsilateral for T2) [35]	Early-stage glottic lesions (T1) rarely metastasize to regional lymph nodes [35]
**Hypopharynx**	Levels II and III [74]	Level IV is considered a secondary level of LNM and Level V is tertiary [74]
**Nasopharynx**	Retropharyngeal nodes (Rouvière nodes) and Levels II–V (particularly II and V) [76]	High propensity for bilateral LNM even in early T-stage tumors; retropharyngeal involvement is common and may occur in isolation.

OSCC: Oral Squamous Cell Carcinoma, OPSCC: Oropharyngeal Squamous Cell Carcinoma.

**Table 2:** Comparative overview of lymph node ratio (LNR) prognostic thresholds across anatomical subsites in head and neck squamous cell carcinoma (HNSCC).

Anatomical Subsite	Reported LNR Cut-Off Range	Prognostic Impact	Study Type/Evidence Level	Key References
Oral Cavity SCC (OSCC)	0.03–0.10 (most frequent 0.05–0.07)	Higher LNR independently associated with worse OS and DFS	Retrospective cohorts/Meta-analyses	[22,97]
Laryngeal SCC	0.06–0.20	Significant association with OS and locoregional control	Retrospective multicenter studies	[35,102]
Oropharyngeal SCC (HPV-negative)	Reported ranges, but heterogeneous	Elevated LNR linked to poorer survival	Retrospective/heterogeneous; limited subsite-specific cut-off standardization	[22,90,103]
Oropharyngeal SCC (HPV-positive)	Not standardized; prognostic effect attenuated/inconsistent	Prognostic impact present but less pronounced than HPV-negative disease	Retrospective/heterogeneous; limited subsite-specific cut-off standardization	[4,22,90]
Hypopharyngeal SCC	0.10–0.20	High LNR associated with aggressive disease and distant metastasis	Institutional series	[89,104]
Nasopharynx SCC	Not consistently defined; limited evidence due to predominance of non-surgical management	LNR may provide additional prognostic stratification in surgically treated or recurrent cases, but data remain limited	Retrospective cohorts and institutional series	[76,105]

**Table 3:** Comparative synthesis of major nodal prognostic markers supporting precision nodal staging in HNSCC.

Marker	Biological/ Pathological Basis	Prognostic Impact	Subsite Differences	HPV Influence	Clinical Implications	Key Refs.
Extranodal extension (ENE)	Tumor spread beyond nodal capsule reflecting aggressive tumor behavior and stromal invasion.	Strong independent predictor of worse OS, DFS, and locoregional control; HR approximately 2–4 across studies.	Consistently adverse across subsites; magnitude varies.	Prognostic impact attenuated but still relevant in HPV-positive OPSCC.	Indication for adjuvant chemoradiotherapy; incorporated into AJCC staging.	[9,11,17]
Number of Positive Lymph Nodes (NPLN)	Reflects metastatic burden and tumor dissemination capacity.	Incremental increase in mortality risk; thresholds around >4–5 nodes associated with worse outcomes.	Particularly relevant in laryngeal and oral cavity SCC.	Higher nodal counts tolerated in HPV-positive disease with less prognostic penalty.	Potential refinement beyond traditional N classification.	[90,91,92]
Lymph Node Ratio (LNR)	Ratio of positive to dissected nodes integrating disease burden and surgical/pathological quality.	Associated with poorer OS and DFS; HR ~1.9–2.4 reported.	Strong evidence in OSCC and advanced laryngeal cancers.	Less clearly defined.	Potential candidate for future TNM refinement.	[22,83,96,97,98,99,100]
Lymph Node Yield (LNY)	Reflects adequacy of neck dissection and pathological evaluation.	Controversial; some studies show survival benefit, others not.	More relevant in OSCC.	Unclear.	Quality indicator rather than pure biological marker.	[115,116,117,118,119]
Nodal Level/Location	Lower neck involvement reflects advanced lymphatic spread.	Associated with increased distant metastasis risk.	Important in hypopharynx and larynx.	Less pronounced.	Radiotherapy planning and prognosis.	[74,93]
Micrometastasis/ITCs	Minimal metastatic burden detected via advanced pathology.	Controversial; potential intermediate-risk category.	Mostly studied in OSCC.	Unknown.	Not yet incorporated into staging.	[1,63,120,121]

## Data Availability

No new data were created or analyzed in this study. Data sharing is not applicable to this article.
